# Evaluating the reflux suppression properties of Gaviscon Infant powder with different milk formulations using an in vitro model of the infant stomach

**DOI:** 10.1038/s41598-025-88638-5

**Published:** 2025-02-08

**Authors:** Fiona McLaughlin, Jeanine Fisher, Mark Atherton, Cathal Coyle

**Affiliations:** 1 Reckitt Healthcare Ltd, Digestive Relief, Dansom Lane, Hull, HU8 7DS UK; 2 Technostics Limited, Research & Development, Daisy Building, Castle Hill Hospital, Cottingham, HU16 5JQ East Yorkshire UK

**Keywords:** Alginate, Cow’s milk allergy, Gastroesophageal reflux, *In vitro* model, Infant formula, Gastrointestinal diseases, Paediatrics

## Abstract

Gastroesophageal reflux (GER) is common in babies and infants and is characterized by effortless regurgitation (with or without vomiting), which is a common symptom of other infant conditions, including cow’s milk protein allergy (CMPA). Various milk formulations are commercially available to help manage GER and CMPA symptoms (extensively hydrolyzed [eHF], partially hydrolyzed [pHF], and amino-acid formulas [AAF]). We aimed to understand if an alginate product (Gaviscon Infant powder) worked as intended in the presence of different milk formulations. A previously evaluated artificial infant stomach model simulated an internal pressure-inducing reflux event (five reflux events performed at 5 min intervals). Suppression activity of Gaviscon Infant combined with eHF, pHF, and AAF was assessed by measuring the height that the refluxate event traveled up a tube mimicking the infant esophagus, alongside a benchmark unhydrolyzed formulation (uHF) with Gaviscon Infant and a negative control without Gaviscon Infant. Each experiment was repeated six times per formulation in a random order. Gaviscon Infant combined with each milk formulation produced a lower height of refluxate versus the negative control. When formulations combined with Gaviscon Infant were grouped by category (eHF, pHF, AAF), two or more products from each category showed a lower refluxate height than the benchmark (uHF). There was a significant difference in median refluxate height (*p* = 0.0011 in Wilcoxon rank sum test) when comparing Gaviscon Infant combined with eHF against Gaviscon Infant combined with pHF, AAF, or uHF (benchmark). Refluxate height did not change significantly between events one and five for each milk formulation combined with Gaviscon Infant. In an artificial infant stomach model, Gaviscon Infant combined with different milk formulations (uHF, eHF, pHF, AAF) produced a lower refluxate height compared with a negative control (uHF without Gaviscon Infant), suggesting that Gaviscon Infant suppresses reflux as intended when used with different milk formulations.

**Supplementary Information:**

The online version contains supplementary material available at 10.1038/s41598-025-88638-5.

## Introduction

Gastroesophageal reflux (GER) is common in infants (less than 12 months of age), affecting at least 40% of this population^1–3^. It is a normal physiological event with no organic cause that involves the involuntary retrograde movement of gastric contents into the esophagus, which can result in effortless regurgitation with or without vomiting^1–4^. Meanwhile, gastroesophageal reflux disease (GERD) in infants occurs when the effect of GER causes troublesome symptoms (e.g. distress, excessive crying, back arching, fussiness during/after feeds, sleep problems) and/or complications, such as faltering growth and respiratory events, which are severe enough to warrant medical treatment^1–4^. Given the overlapping symptoms of GER and GERD, the two are often considered as one spectrum condition, rather than distinct conditions^5,6^. There is overlap between the symptoms of GERD and cow’s milk protein allergy (CMPA), and studies have reported the presence of CMPA in 16–56% of infants with symptoms attributed to GERD^7^.

Regurgitation is a gastrointestinal sign that has been shown to occur daily in up to 70% of healthy infants (less than 12 months of age)^8^, and the global prevalence is reported to be 30% based on the Rome III criteria published in 2006 (two or more episodes of regurgitation per day for at least 3 weeks)^9,10^. When the frequency and severity of regurgitation is at the upper end of the spectrum, it can be distressing for the infant due to pain and discomfort and stressful for parents/carers^11–13^.

Typically, regurgitation becomes less frequent with time (resolving in 90% of affected infants before 1 year of age), and does not usually need further investigation or treatment^1^. However, treatment options are available if regurgitation is associated with marked distress, and non-pharmacological interventions are typically used first-line^1,14^. Feed thickeners are commonly recommended to manage symptoms^1,14^, despite the fact that only a small number have demonstrated a meaningful reduction in regurgitation episodes^15^. It is assumed that they increase the viscosity and density of liquid, enabling food to be retained in the stomach^16^. In addition, alginates (natural polysaccharide polymers isolated from brown seaweed), which are currently available in a few countries (e.g. United Kingdom [UK]), are recommended as a second-line therapy if feed thickeners have not provided adequate symptom relief^1,17,18^. Alginates have been proven to reduce the number of reflux episodes to a greater extent than feed thickeners^15^. Guidelines recommend that if feed thickeners are not successful, then they should be stopped, and alginate therapy can be offered for a trial period of 1 to 2 weeks. If successful, alginate therapy can be continued and then stopped at regular intervals to assess if the infant has recovered^1^. If alginates are not available, acid suppressive techniques or prokinetics are considered the next option following the unsuccessful use of feed thickeners^14^. However, given that acid does not play a major role in infant GER^19^, these treatments are not considered appropriate in most cases of infant reflux and should only be reserved for infants with diagnosed acid related GERD^14^.

Gaviscon Infant is a powder formulation that contains two alginates: sodium alginate and magnesium alginate (225 mg and 87.5 mg, respectively, per unit dose), and is indicated for gastric regurgitation and GER in infants and young children^20^. Gaviscon Infant works by reacting with acidic gastric contents to form a viscous gel; calcium ions and milk proteins in infant milk formulations promote the cross-linking of alginate polymers, increasing the viscosity and causing gelling and thickening of the milk formulation in the stomach, thus reducing the chance of retrograde movement of gastric contents into the esophagus^5,20,21^. As the mode of action of Gaviscon Infant is physical, it is not expected that it would modulate the immunological or inflammatory response.

Different milk formulations, such as extensively hydrolyzed formulas (eHF), partially hydrolyzed formulas (pHF), and amino-acid formulas (AAF), are commonly used in the management of reflux, CMPA, and other infant complaints, such as colic^22,23^. To the best of our knowledge, the effects of different milk formulations on the reflux suppressive properties of Gaviscon Infant have not been studied previously. Evaluating the use of Gaviscon Infant in combination with different milk formulations may provide confidence in the efficacy of the product, and offer value in the guidance of dual therapy for the conditions mentioned.

Research within the pediatric population is vital to increase our knowledge of the effects of medicines and for the optimization of care^24,25^; however, there are ethical factors associated with pediatric clinical studies^25,26^. There is great value in conducting research within the laboratory setting to evaluate products that may be suitable for the treatment of GER in infants, before exposing young vulnerable patients to a clinical trial. To aid with this, a simplified in vitro artificial infant stomach model was recently developed to simulate internal reflux and measure the height and amount of refluxate traveling up the infant esophagus and was evaluated for reproducibility and repeatability^5^. The simplified model does not mimic the full in vivo environment; however, it allows quick and reproducible comparison of reflux suppression properties of Gaviscon Infant in combination with different milk formulations.

The current study evaluated the performance robustness of Gaviscon Infant powder using the artificial infant stomach model. The primary objective was to evaluate whether the reflux suppression properties of Gaviscon Infant powder work as intended in the presence of different commercially available milk formulations that are typically used in the management of CMPA and other infant digestive complaints compared with a standard unhydrolyzed milk formulation without Gaviscon Infant.

## Materials and methods

### Formulations tested

In total, seven milk formulations from the UK, seven from Latin America (LATAM), and two from the United States (US) were tested in combination with Gaviscon Infant powder (anti-reflux formulation) alongside a benchmark UK SMA Pro formulation (unhydrolyzed formulation [uHF]) in combination with Gaviscon Infant and a negative control of SMA Pro with no Gaviscon Infant added. The milk formulations were categorized based on the extent of the hydrolysis of the cow’s milk proteins present in the product: pHF, eHF, or AAF (Table 1).

**Table 1:** Overview of milk formulations used in performance testing.

Market	Product	Category
UK	Puramino (UK)	Amino acid formulation
	Similac Alimentum	Extensively hydrolyzed formulation
	Aptamil Pepti	Extensively hydrolyzed formulation
	SMA Althera	Extensively hydrolyzed formulation
	Aptamil Comfort	Partially hydrolyzed formulation
	Cow & Gate Comfort	Partially hydrolyzed formulation
	SMA Comfort	Partially hydrolyzed formulation (thickened)
	SMA Pro (UK)*	Unhydrolyzed formulation
LATAM	Alfamino HMO	Amino acid formulation
	EleCare	Amino acid formulation
	Neocate LCP	Amino acid formulation
	Puramino (LATAM)	Amino acid formulation
	Alfaré HMO	Extensively hydrolyzed formulation
	Novamil Allernova	Extensively hydrolyzed formulation
	Novamil Rice	Extensively hydrolyzed formulation
US	Nutramigen 1 with LGG Hypoallergenic Formula	Extensively hydrolyzed formulation
	Enfamil Neuro Pro Gentlease	Partially hydrolyzed formulation (thickened)

*Product used in combination with Gaviscon Infant as a benchmark for UK, LATAM, and US milk formulations.

HMO, human milk oligosaccharides; LATAM, Latin America; LCP, long-chain polyunsaturated; LGG, *Lactobacillus rhamnosus* GG; UK, United Kingdom; US, United States.

### Experimental method

The artificial infant stomach model has been described previously^5^. The model was designed and developed by Technostics Limited, was used to simulate an internal pressure-inducing reflux event. The model has previously been evaluated by three independent operators to demonstrate its robustness, reproducibility, and ease of use, and it allows simulation of internal reflux and measurements of the height and amount of gastric refluxate traveling up an artificial esophagus^5^.

**Fig. 1:**
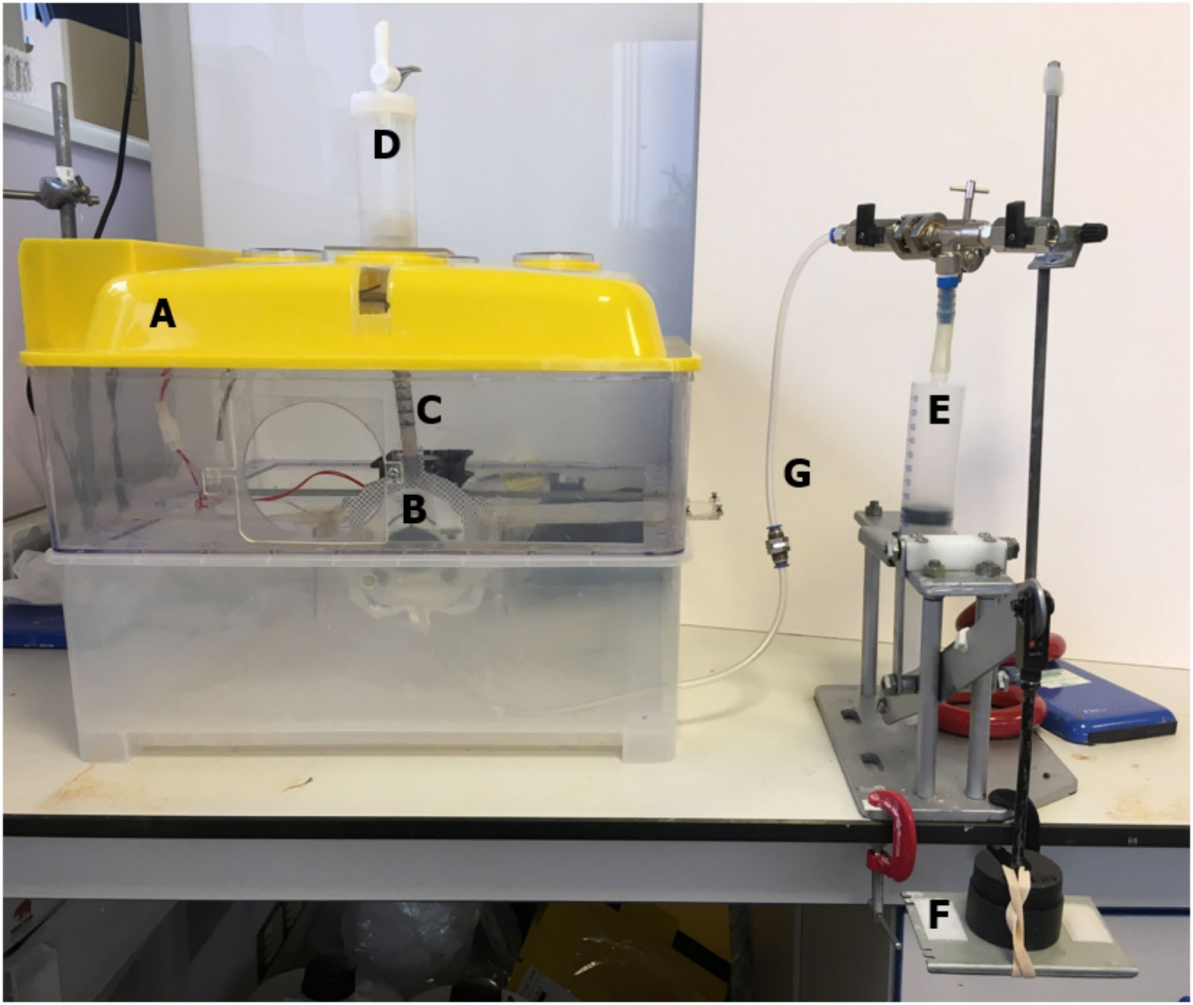
Artificial stomach model. (**A**) Heated (37 °C) incubation unit; (**B**) Infant artificial stomach (85 mL); (**C**) Infant esophagus (12 cm) marked with 1 cm increments. Refluxate height recorded; (**D**) Reflux collection vessel. Volume measured; (**E**) Air via 100 mL syringe; (**F**) Force provided by 0.3 kg weight; (**G**) Creation of reflux event connected to bottom of stomach by one- way valve. Image taken from Fisher et al. *Drugs R D* 2021;21:331–9, used under CC BY 4.0 (https://creativecommons.org/licenses/by-nc/4.0/), cropped from original.

The artificial stomach model and its contents were housed in a heated incubation unit to maintain a consistent temperature (37 °C) throughout the experiments. The model was composed of a 150 mL infant urinary drainage bag purchased from Great Bear Healthcare, UK (Product code: 10125B) as the artificial stomach with a test liquid volume of ~ 85 mL, which reflects the lower end of a normal feed volume (90–120 mL) of a 1-month-old (youngest age to be prescribed Gaviscon Infant)^27^. The urinary drainage bag had two openings at opposite ends, one of which has a built-in one-way valve, allowing liquid or gas into the bag but not out again. The bag was mounted, so that the valve was positioned at the bottom, which prevented the loss of stomach contents whilst allowing a simulated reflux event to be introduced via the connected reflux event apparatus. A 100 mL syringe was connected to the artificial stomach, enabling air to be forced into the stomach to simulate a reflux event, while a standardized reproduceable air pressure and volume was achieved by releasing a 0.3 kg weight levering the plunger through the syringe. To connect the reflux event apparatus to the artificial stomach, flexible silicone tubing (6 mm internal diameter × 8 mm outer diameter) was used. The opening at the top of the urinary drainage bag did not have a valve and was directly connected to 12 cm long silicone tubing (8 mm internal diameter; 10 mm outer diameter) to mimic the infant esophagus (marked with 1 cm increments to allow reflux activity to be measured) (Fig. 1).

The in vitro model was designed to quickly and reproducibly compare the reflux suppression properties of Gaviscon Infant in combination with different milk formulations compared with a standard unhydrolyzed milk formulation without Gaviscon Infant added, rather than mimicking the full in vivo environment (e.g. considering gastric digestion, peristalsis with physiological pressure, physiological size and closure of the cardia and pylorus). Model variables were therefore fixed to provide a ‘worst case’ environment, where Gaviscon Infant would be most challenged.

Milk formulations were prepared in accordance with their individual instructions for use. In brief, each milk formulation was prepared by adding the appropriate amount of formula to 120 mL of 37 °C tap water in a bottle and shaking until fully dissolved. One sachet of Gaviscon Infant (0.65 g) was then added to the bottle and shaken well. The contents were transferred to a glass beaker, placed onto a heated stirrer, and 1 M HCl was used to adjust the pH to 4.8, with the temperature maintained at 37 °C. We were interested in the initial pH in which the gel forms and thickens in the stomach; therefore, a pH of 4.8 was chosen, as this sits within the physiological range observed during first feeding^28,29^. Full details on formulation preparation can be found in the “Electronic Supplementary Material” (Table 1).

Once the milk formulation was prepared, 85 mL was added to the artificial stomach. A reflux event was created by forcing 100 mL of air through the stomach. The maximum distance that the milk traveled up the 12 cm tube (which was used to mimic the esophagus), was recorded as the refluxate height. During each experiment (first experiment performed: January 2022; last experiment performed: April 2022), five reflux events were performed at 5 min intervals. Each experiment was repeated six times for each milk formulation in a random order. During each experiment the artificial stomach and its contents were kept in an environment of 37 °C.

### Statistical analysis

Statistics were performed using GraphPad Prism 9 for Windows 64-bit Version 9.5.0 (730) and SAS v9.4. The primary outcome variable of the study was refluxate height measured in centimeters, which is a continuous variable that is measured on a scale with infinite plausible outcomes. The assumption of the distribution of the data was initially tested using a Shapiro–Wilk test for normality (*p* < 0.0001). As the outcome variable did not meet this assumption and the refluxate heights were found to be non-normally distributed, a non-parametric equivalent test (Wilcoxon rank sum test), which does not require data to follow a normal distribution, was performed to determine if there was any significant difference between the refluxate height observed when Gaviscon Infant was used with each of the four formulation categories (uHF, pHF, eHF, AAF). For each formulation, the height observed from each individual reflux event (*n* = 5) of each replicate (*n* = 6) was included in the rank test; therefore, *n* = 30 observations per formulation were included in each category grouping. The alpha level for all hypothesis testing was set at an industry standard of α = 0.05, meaning if the p-value was < 0.05, we could say with 95% confidence that the results were not random, nor by chance, but show a true significant difference.

## Results

### Ranked performance

**Fig. 2:**
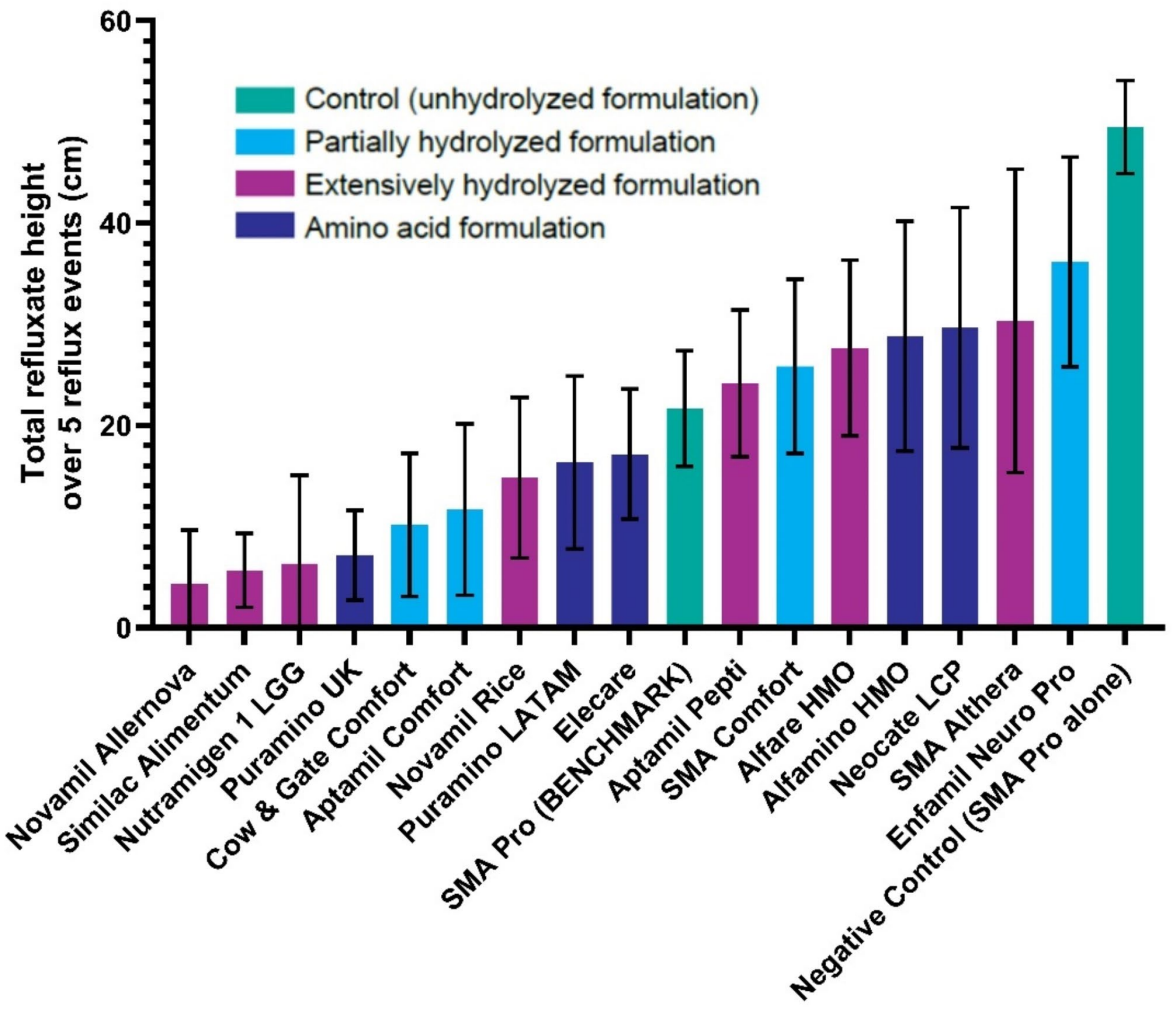
Ranked performance of Gaviscon Infant (from most to least effective at suppressing reflux; *n* = 6, mean ± SD) according to formulation type for reflux events combined. HMO, human milk oligosaccharides; LATAM, Latin America; LCP, long-chain polyunsaturated; LGG, *Lactobacillus rhamnosus* GG; SD, standard deviation; UK, United Kingdom.

At each of the five reflux events, Gaviscon Infant in combination with each milk formulation produced a lower height of refluxate compared with the negative control without Gaviscon Infant added to it. As the refluxate height was evenly distributed over each event, the five reflux events were summed and the performance of Gaviscon Infant in combination with each formulation (categorized by formulation type [eHF, pHF, AAF]) was ranked according to total refluxate height. Each formulation category was spread across the rankings; however, at least one formulation from each category outperformed the benchmark (unhydrolyzed formula) (Fig. 2).

### Performance across reflux events

**Fig. 3:**
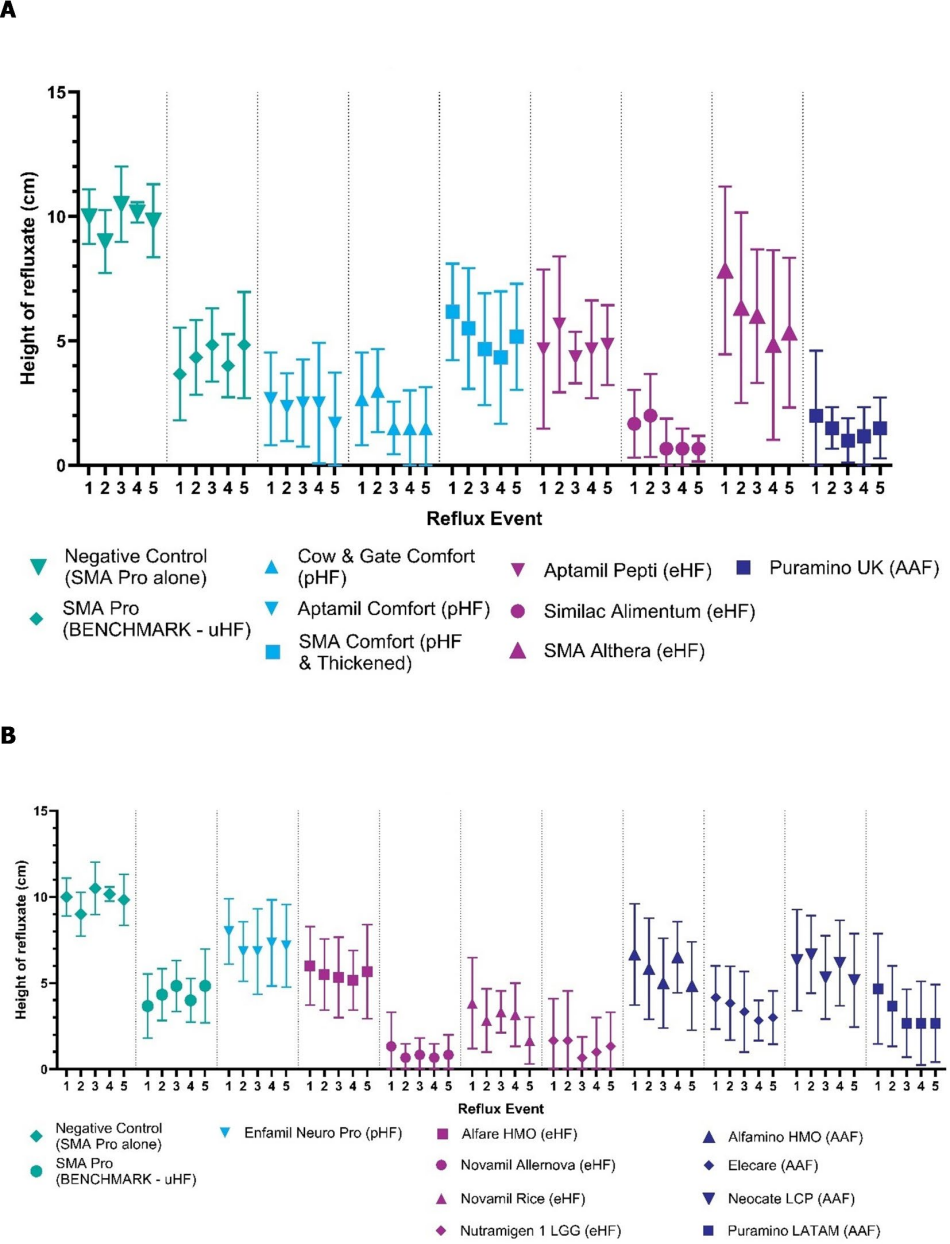
Performance of Gaviscon Infant for all reflux events (*n* = 6, mean ± SD) with each UK milk formulation (**A**) and each LATAM/US milk formulation (**B**). AAF, amino acid formulation; eHF, extensively hydrolyzed formulation; HMO, human milk oligosaccharides; LATAM, Latin America; LCP, long-chain polyunsaturated; LGG, *Lactobacillus rhamnosus* GG; pHF, partially hydrolyzed formulation; SD, standard deviation; UK, United Kingdom; uHF, unhydrolyzed formulation.

The height of refluxate at each reflux event did not change significantly between events one and five for all milk formulations (UK, LATAM, and US products) in combination with Gaviscon Infant. Gaviscon Infant in combination with each milk formulation produced a lower height of refluxate than the negative control, while the performance of Gaviscon Infant was related to the milk formulation used (Fig. 3A,B).

### Comparison of statistical significance

**Fig. 4:**
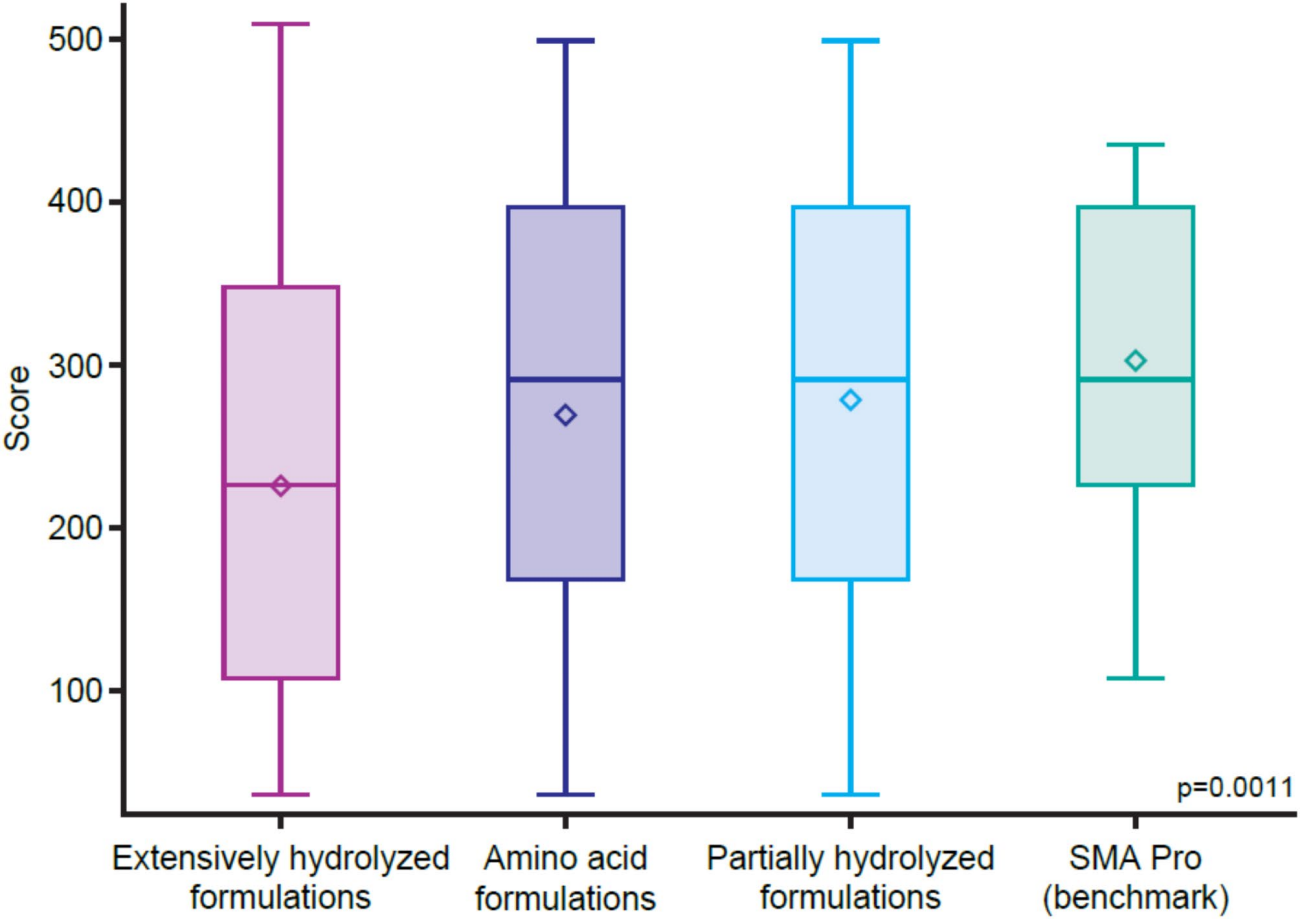
Distribution of Wilcoxon Scores for refluxate height for each milk formulation category. Boxplot interpretation: central line (median) represents the median value of the dataset; box edges represent the interquartile range and the lower and upper edges of the box indicate the first and third quartiles, respectively, and this range captures the middle 50% of the data; whiskers extend from the box to the smallest and largest values within 1.5 times the interquartile range from the quartiles, showing the spread of the data; a non-parametric Wilcoxon rank sum test was used to compare the medians of the four independent samples, and the p-value indicates the statistical significance of the difference between the four groups.

A Wilcoxon rank sum test was performed to compare the refluxate height for Gaviscon Infant combined with each formulation category (eHF, pHF, AAF, uHF). A statistically significant difference (*p* = 0.0011) in median refluxate height was observed when comparing Gaviscon Infant with eHF against Gaviscon Infant in combination with pHF, AAF, or uHF (benchmark) (Fig. 4).

## Discussion

Regurgitation is experienced by a large proportion of healthy infants^8^, and is a common symptom of GER and other infant conditions, including CMPA^7,30^. Typically, regurgitation does not need further investigation and treatment^1^; however, for infants suffering from regurgitation associated with marked distress, appropriate treatment options are needed to reduce associated pain and discomfort^1,11^. Alginate-based formulations are a potential therapeutic option for infants with reflux and regurgitation^7,31^, and are recommended in some markets as part of the treatment pathway^1,18^. In addition, meta-analyses have demonstrated the ability of alginates to reduce the number of daily regurgitation episodes^12,32^.

With numerous milk formulations on offer^33^, including those designed specifically for infant conditions where regurgitation is a common symptom (e.g. CMPA)^7,30^, it is important to investigate the effectiveness of reflux suppression treatment options in the presence of different milk formulations. Our study aimed to investigate whether Gaviscon Infant, an alginate-based formulation indicated for gastric regurgitation and GER in infants (not recommended for infants with intestinal obstructions)^20^, worked as intended in the presence of commercially available milk formulations (eHF, pHF, AAF). We utilized an in vitro artificial infant stomach model to simulate internal reflux and measure the height and amount of refluxate traveling up the infant esophagus^5^. The model does not account for interactions between various body processes and cellular biochemistry and has not been validated in comparison to in vivo studies. However, it has previously been evaluated for reproducibility and repeatability^5^, and allows initial laboratory research to be conducted without the need for a clinical trial. This is particularly valuable, as performing research in the pediatric population is ethically challenging^25,26^.

Our results build upon previous work by Fisher et al., which demonstrated that the in vitro model could discriminate between feeds of a standard unhydrolyzed formulation with and without the addition of Gaviscon Infant^5^. Reflux is common in the infant stomach after feeding due to physiological reasons; reducing refluxate height is beneficial as it results in fewer uncomfortable symptoms. In our study, Gaviscon Infant, a product intended to reduce refluxate height, in combination with each milk formulation produced a lower height of refluxate versus the negative control (standard uHF [SMA Pro] without Gaviscon Infant). It is likely that the lower refluxate heights observed in our study are due to the presence of Gaviscon Infant coupled with the properties of the milk formulation. However, as the study did not include an individual control for each milk formulation, it cannot be said with certainty that the refluxate heights measured in vitro were influenced by Gaviscon Infant and to what extent. It is important to highlight that some interactions between Gaviscon Infant and milk formulations included in the study, resulted in an extremely low refluxate height compared with the negative control. This is probably the result of increased viscosity, as previously demonstrated^21^, which is likely followed by slowed emptying into the stomach, causing elongated contact of refluxate with regions of the esophagus. Given that most episodes of infant reflux are weakly acidic or non-acidic^6^, increased retention time is unlikely to cause the same level of mucosal damage that is observed in cases of adult reflux^34^. It should also be noted that the strong efficacy in preventing reflux can, in very rare cases, lead to bezoar formation^35^, although this is noted to occur seldomly^36^.

Our study also revealed that Gaviscon Infant in combination with each formulation category outperformed the unhydrolyzed benchmark formulation. However, statistical analyses showed that when the milk formulations were grouped according to formulation category (eHF, pHF, AAF) and compared against each other, Gaviscon Infant in combination with eHF performed the best with respect to mean refluxate height. As Gaviscon Infant works via promotion of cross-linking by calcium ions and milk proteins present in the feed^20^, it was expected that the product would have worked least well in combination with eHF, where milk proteins are broken down. It appears that the broken-down nature of the proteins helped the performance of Gaviscon Infant, potentially because smaller milk peptides could be more effective at promoting alginate cross-linking than larger milk peptides or proteins. These smaller peptides in eHF may not interact with alginates in the same way as larger proteins in partially hydrolyzed or intact protein formulas. This may lead to differences in the thickness and stability of the gel formed, potentially improving the efficacy of the alginates in managing reflux; however, the mechanism is unknown and the results cannot be considered conclusive from one in vitro assessment. While direct interaction between eHF and alginates may be occurring, the formula itself may also have an impact on refluxate height given eHF without alginates is a recommended non-pharmacologic intervention for treating infant reflux^37^. We also found that the height of refluxate did not change significantly between events one and five for all milk formulations in combination with Gaviscon Infant. This indicates that the reflux suppression action of Gaviscon Infant occurs within 5 min and is sustained throughout the 25 min experiment despite the physical disturbance of each reflux event.

Although our results cannot be directly extrapolated to the in vivo situation, they add to the existing body of evidence, which demonstrates the effect of Gaviscon Infant in vivo in infants with symptoms of GER. A study by Del Buono et al.^21^investigated the influence of Gaviscon Infant on GER in infants versus placebo using pH and intraluminal impedance measurements, which allowed for detection of both acid and non-acid reflux episodes. Of the measurements, a marginal but significant difference between Gaviscon Infant and placebo in average refluxate height was demonstrated. In addition, Corvaglia et al. found that Gaviscon Infant lowered the number of GER events reaching the proximal esophagus^38^. Furthermore, studies by Buts et al., Miller et al., and Salvatore et al. demonstrated a reduction in the number of episodes of regurgitation compared with placebo^7,39,40^. Salvatore et al. also found that alginate administration resulted in a significant reduction in crying-fussiness, cough, and reflux episodes^7^, while a study by Le Luyer et al. 1992 demonstrated a significant reduction in the frequency of regurgitation and vomiting^41^. Taken together these results suggest a role for alginates in the management of GER symptoms, as an alternative to feed thickeners if unsuccessful, and acid-suppressing drugs (e.g. proton pump inhibitors), which are increasingly used in situations that do not adhere to management guidelines and in spite of their potential side effects^1,14,31^.

Our study takes a step towards understanding how Gaviscon Infant works with different milk formulations; however, it is important to highlight limitations of the study and areas for future research. Although the correct number of replicates were run for each milk formulation (*n* = 6), the study was considered to be of a small scale and with limited statistical power. Studies with a larger number of replicates would have the potential to provide more robust data. In addition, non-parametric statistical methods were used to analyse the data, which are less sensitive than parametric methods particularly when sample sizes are small. The data from our study did not follow a normal distribution and it was not possible to use parametric methods. A Wilcoxon rank sum test was, therefore, performed and indicated statistically significant findings (*p* > 0.001). Performance robustness testing demonstrated a difference between Gaviscon Infant and all milk formulations versus the negative control (SMA pro without Gaviscon Infant). However, negative controls were not included for other milk formulations, and this limitation means it is not certain whether the reductions in refluxate height were due to the effects of Gaviscon Infant or due to the properties of the milk formulation. Further investigation is required to better understand the full effect of Gaviscon Infant. In addition, measurement of the viscosity of different milk formulations (with and without Gaviscon Infant) would lead to a better understanding of formula–treatment interactions, potentially allowing for rapid screen testing to determine which combinations allow for the most effective reflux suppression. Moreover, as the in vitro model used was simplified and did not reflect the full in vivo environment, the performance of Gaviscon Infant with different milk formulations may be affected by physiological conditions and variables in vivo that could not be reflected in vitro. Given the challenges in assessing reflux in vivo in infants, it would be beneficial to conduct further in vitro testing in a validated dynamic stomach model. Finally, we found that Gaviscon Infant in combination with eHF performed the best with respect to refluxate height, which was an unexpected outcome and one that warrants further investigation.

## Conclusion

A simplified in vitro model of the infant stomach was used to simulate internal reflux and measure the height and amount of refluxate traveling up an artificial esophagus, which allowed the primary aim of the study to be evaluated. Even when used with a variety of products that had substantially different formulations, Gaviscon Infant worked as intended and produced a lower height of reflux compared with a negative control. The study provides initial evidence to suggest that Gaviscon Infant could be recommended for the management of GER in infants and be used alongside a variety of milk formulations, including those that are often used in the treatment of CMPA and other digestive complaints (pHF, eHF, and AAF).

## Electronic supplementary material

Below is the link to the electronic supplementary material.

Supplementary Material 1

## Data Availability

All data supporting the findings of this study are available within the paper and its Electronic Supplementary Material.
