# Supplementary material for: Evaluating the reflux suppression properties of Gaviscon Infant powder with different milk formulations using an in vitro model of the infant stomach
Source: Sci Rep. 2025 Feb 8;15:4787. doi: 10.1038/s41598-025-88638-5 (PMC11807092; doi:10.1038/s41598-025-88638-5)
Supplement: Supplementary file 1 — Supplementary Material 1 [file 41598_2025_88638_MOESM1_ESM.docx]

**SUPPLEMENTARY FILE 1**

**Evaluating the reflux suppression properties of Gaviscon Infant powder with different milk formulations using an in vitro model of the infant stomach**

**Authors:** Fiona McLaughlin,^1^ Jeanine Fisher,^2^ Mark Atherton,^2^ Cathal Coyle^1^

*^1^Reckitt Healthcare Ltd, Digestive Relief, Dansom Lane, Hull, HU8 7DS, UK; ^2^Technostics Limited, Research & Development, Daisy Building, Castle Hill Hospital, Cottingham, East Yorkshire, HU16 5JQ, UK*

***Corresponding author:** Fiona McLaughlin, BSc

Reckitt Healthcare Ltd., Digestive Relief, Dansom Lane, Hull, HU8 7DS, UK

**Email:** [Fiona.Burke@reckitt.com](mailto:Fiona.Burke@reckitt.com)

**Supplementary Table S1.** Milk formulation preparation for performance experiments

| **Products** | **Preparation instructions** |
| --- | --- |
| Alfaré HMO  Novamil Allernova  Novamil Rice  Puramino (LATAM)  Aptamil Pepti  Nutramigen 1 with LGG | 1) Record mass (~4.5 g) of one levelled scoop of formulation x3  2) Add four scoops to 120 mL of 37°C tap water in a bottle  3) Shake bottle well until fully dissolved  4) Mix in a sachet of Gaviscon Infant to the bottle and shake well  5) Transfer into a glass beaker, place onto a heater stirrer, and adjust to pH 4.80 with 1M HCl, maintaining a temperature of 37°C |
| Alfamino HMO | 1) Record mass (~4.43 g) of one levelled scoop of formulation x3  2) Add four scoops to 120 mL of 37°C tap water in a bottle  3) Shake bottle well until fully dissolved  4) Mix in a sachet of Gaviscon Infant to the bottle and shake well  5) Transfer into a glass beaker, place onto a heater stirrer, and adjust to pH 4.80 with 1M HCl, maintaining a temperature of 37°C |
| Neocate LCP  Aptamil Comfort  Cow & Gate Comfort Milk | 1) Record mass (~4.6 g) of one levelled scoop of formulation x3  2) Add four scoops to 120 mL of 37°C tap water in a bottle  3) Shake bottle well until fully dissolved  4) Mix in a sachet of Gaviscon Infant to the bottle and shake well  5) Transfer into a glass beaker, place onto a heater stirrer, and adjust to pH 4.80 with 1M HCl, maintaining a temperature of 37°C |
| EleCare | 1) Record mass (~9.4 g) of one levelled scoop of formulation x3  2) Add two scoops to 120 mL of 37°C tap water in a bottle  3) Shake bottle well until fully dissolved  4) Mix in a sachet of Gaviscon Infant to the bottle and shake well  5) Transfer into a glass beaker, place onto a heater stirrer, and adjust to pH 4.80 with 1M HCl, maintaining a temperature of 37°C |
| Similac Alimentum  SMA Comfort | 1) Record mass (~4.2 g) of one levelled scoop of formulation x3  2) Add four scoops to 120 mL of 37°C tap water in a bottle  3) Shake bottle well until fully dissolved  4) Mix in a sachet of Gaviscon Infant to the bottle and shake well  5) Transfer into a glass beaker, place onto a heater stirrer, and adjust to pH 4.80 with 1M HCl, maintaining a temperature of 37°C |
| SMA Althera  Puramino (UK) | 1) Record mass (~4.4 g) of one levelled scoop of formulation x3  2) Add four scoops to 120 mL of 37°C tap water in a bottle  3) Shake bottle well until fully dissolved  4) Mix in a sachet of Gaviscon Infant to the bottle and shake well  5) Transfer into a glass beaker, place onto a heater stirrer, and adjust to pH 4.80 with 1M HCl, maintaining a temperature of 37°C |
| Enfamil Neuro Pro Gentlease | 1) Record mass (~8.7 g) of one levelled scoop of formulation x3  2) Add two scoops to 120 mL of 37°C tap water in a bottle  3) Shake bottle well until fully dissolved  4) Mix in a sachet of Gaviscon Infant to the bottle and shake well  5) Transfer into a glass beaker, place onto a heater stirrer, and adjust to pH 4.80 with 1M HCl, maintaining a temperature of 37°C |
| SMA Pro (negative control) | 1) Record mass (~9 g) of one levelled scoop of formulation x3  2) Add two scoops to 120 mL of 37°C tap water in a bottle  3) Shake bottle well until fully dissolved  4) Transfer into a glass beaker, place onto a heat stirrer, and adjust to pH 4.80 with HCI, maintaining a temperature of 37°C |
